# Recognition and Treatment of Tardive Dyskinesia in Individuals with Intellectual Disability

**DOI:** 10.1155/2020/8886980

**Published:** 2020-12-11

**Authors:** Robert O. Morton, Lucas C. Morton, Rissa Fedora

**Affiliations:** ^1^Rolling Hills Hospital, Ada, OK, USA; ^2^Oklahoma State University College of Osteopathic Medicine, Tulsa, OK, USA; ^3^University Hospital and Medical Center, Tamarac, FL, USA

## Abstract

Individuals with intellectual disability (ID) commonly suffer from comorbid psychiatric and behavioral disorders that are frequently treated by antipsychotic medications. All individuals exposed to first- and second/third- generation antipsychotics are at risk for developing tardive dyskinesia (TD), characterized by abnormal, involuntary movements of the mouth/tongue/jaw, trunk, and extremities. TD can be highly disruptive for affected individuals and their caregivers, causing embarrassment, isolation, behavioral disturbances, and reduced functioning and quality of life. Information on TD incidence in individuals with ID is limited, but 2 small US studies reported TD prevalence rates of 42-45% in inpatients with ID. The safety and efficacy of vesicular monoamine transporter type 2 (VMAT2) inhibitors approved for treatment of TD in adults have been demonstrated in multiple clinical trials, but they excluded individuals with ID. Clinical characteristics and treatment outcomes of 5 adults (aged 28–63 years) with mild-to-severe ID and TD are presented, illustrating TD symptoms before/after treatment. All individuals had multiple comorbid psychiatric, behavioral, and other medical conditions, history of antipsychotic exposure, and abnormal movements affecting the tongue/mouth/jaw (*n* = 5), upper extremities (*n* = 5), lower extremities (*n* = 3), and trunk (*n* = 2), resulting in diminished ability to speak (*n* = 2), ambulate (*n* = 3), and perform activities of daily living (*n* = 3). Treatment with valbenazine resulted in meaningful improvements in TD symptoms and improved daily functioning, demeanor, and social/caregiver interactions. Given the high likelihood of antipsychotic exposure in the ID population, it is appropriate to screen for TD at every clinical visit through careful monitoring for abnormal movements and questioning the individual/caregiver regarding abnormal movements or TD-related functional impairments (i.e., speaking, swallowing, eating, ambulating, and social functioning). In this study, 5 individuals with ID and TD received once-daily valbenazine and experienced marked improvement in TD symptoms and daily functioning, resulting in increased quality of life for affected individuals and caregivers.

## 1. Introduction

Intellectual disability (ID) is characterized by significant limitations in cognitive functioning (i.e., learning, reasoning, and problem solving) and adaptive behaviors (i.e., communication, practical skills, and independent living) that originate before the age of 18 years [1]. Depending on the population and level of severity of ID assessed, prevalence estimates have varied from 1-3% [2, 3]. Individuals with ID commonly suffer from comorbid psychiatric disorders such as depression, schizophrenia, and bipolar disorder and present with challenging behaviors such as aggression, self-injury, or inappropriate social conduct. Antipsychotic medications, both first- and second/third- generation, are frequently used in these individuals to manage these psychiatric and behavioral issues [4–8]. In a Canadian population-based study of approximately 51,000 adults with ID, 39% received antipsychotic treatment, which increased to 56% in individuals living in group homes [7]. In a UK cohort of approximately 33,000 adults with ID, 28% were treated with antipsychotics [8].

All individuals who are exposed to antipsychotics (both first- and second/third- generation) are at risk for developing tardive dyskinesia (TD), a movement disorder characterized by abnormal and involuntary choreoathetoid (irregular, dancelike, and/or slow writhing) movements of the mouth, tongue, jaw, trunk, and extremities [9, 10]. According to The Diagnostic and Statistical Manual of Mental Disorders, 5th Edition (DSM-5), TD typically appears after at least a few months of antipsychotic use and may develop even sooner in older individuals [11]. However, there is no “safe” minimum period, as TD can appear after weeks or even days of antipsychotic exposure [12]. The symptoms of TD are persistent and can range in severity from mild to disabling or even life-threatening [13, 14]. Without proper treatment, remission is unlikely, even when antipsychotic medications are reduced or withdrawn [15, 16].

While second-generation antipsychotics were initially thought to be associated with reduced risk of TD, growing evidence indicates that TD risk is significant for both second- and first-generation antipsychotics [17–20]. In a recent meta-analysis, the prevalence of TD among the general population was 30% in those receiving first-generation antipsychotics, 21% in those receiving second-generation antipsychotics, and 7% in first-generation naïve individuals receiving second-generation antipsychotics [17]. There is limited information regarding the incidence or prevalence of TD in individuals with ID, but a recent UK population-based cohort study provided evidence supporting the long-held assumption that people with ID are more susceptible to antipsychotic-induced movement disorders [21], and 2 small US inpatient studies reported TD prevalence rates of 42-45% in individuals with ID [22, 23].

Until recently, TD treatment options were limited to antipsychotic tinkering, off-label uses of medications (e.g., anticholinergics, which can exacerbate TD), and herbal supplements, all of which have weak or limited evidence of efficacy [24, 25]. In April 2017, a novel vesicular monoamine transporter type 2 (VMAT2) inhibitor, valbenazine, was approved in the US for the treatment of adults with TD. Valbenazine, a valine ester of a highly selective isomer of tetrabenazine ([+]-*α*-dihydrotetrabenazine), has demonstrated safety and efficacy in double-blind, placebo-controlled clinical trials and is considered (along with deutetrabenazine, a deuterated molecular form of tetrabenazine) the first-line therapy for treatment of TD; however, individuals with ID were excluded from the trials [24, 26–29].

Appropriate recognition and treatment of TD in individuals with ID are essential, as TD symptoms can be highly disruptive for the affected individuals as well as their caregivers, causing embarrassment, isolation, increased behavioral disturbances, and reduction in daily functioning and quality of life. To help improve awareness of the clinical presentation and management of TD in individuals with ID, the current case series describes the clinical characteristics, symptoms, and outcomes of 5 individuals with ID who were diagnosed with TD and treated with once-daily valbenazine.

## 2. Case Presentations

### 2.1. Case 1

A 63-year-old nonverbal male with moderate ID who lives in a group home had a history of multiple significant comorbid diagnoses, including arthritis, anemia, gastroesophageal reflux disease (GERD), hyperlipidemia, and Parkinson's disease (Table 1). In addition, the patient was diagnosed with major depressive disorder and had been treated for several years with the atypical antipsychotic, quetiapine (300 mg once daily (QD)).

TD symptoms included constant tongue protrusion and intermittent chewing movements of the jaw that led to severe drooling and difficulty swallowing. The patient also had excessive, pronounced eye blinking, bilateral shoulder/hand/finger movements (predominant on the left side at rest), and constant left foot tapping. His abnormal body movements affected his balance and stability such that he could not stand stably without support and required a wheelchair for mobility (Table 2). In addition, his abnormal movements caused difficulty performing activities of daily living (ADLs), such as feeding himself. The duration of these symptoms is unknown. The patient was initiated on valbenazine (40 mg every night at bedtime (QHS) for 1 week, discontinued for 1 week due to lack of insurance, then resumed at 80 mg QHS) in April 2018. No other changes were made to the medication regimen at the time of valbenazine initiation. After 2 months of valbenazine treatment, the patient had no tongue protrusion or chewing movements and was able to close his mouth, which led to decreased drooling and improved swallowing. His eye blinking was less frequent and less pronounced, and he had minimal shoulder/hand/finger movements and no foot tapping. The decrease in abnormal movements led to improved stability and posture such that he could stand and ambulate with a walker (Table 2). Additionally, he was able to feed himself independently and was more interactive with caregivers. The patient remains stable on valbenazine (80 mg QHS) with no changes to his medication regimen and no reported adverse events (Table 1).

### 2.2. Case 2

A 63-year-old female with mild ID who lives at home with caregivers had a history of multiple significant comorbid diagnoses, including type 2 diabetes, fibromyalgia, GERD, hyperlipidemia, hypertension, hyperthyroidism, overactive bladder, and neuropathy (Table 1). In addition, the patient was diagnosed with schizoaffective disorder, bipolar type, and had prior long-term exposure to the atypical antipsychotics, olanzapine (20 mg QD) and risperidone (3 mg QD).

TD symptoms were repetitive tongue protrusion, lip smacking, and chewing movements of the mouth/jaw that led to unclear speech and difficulty communicating. The patient also had constant bilateral arm/hand/finger movements which caused instability during ambulation and led to the need for wheelchair assistance (Table 2). Her abnormal movements, especially the tongue protrusions, had caused her to become very self-conscious and isolated. The exact duration of the TD symptoms is unknown, but the patient reported having them for at least 4 years. She was initiated on valbenazine (40 mg QHS for 1 week, then escalated to 80 mg QHS) in June 2017. At the same time that valbenazine was initiated, olanzapine and risperidone were discontinued due to parkinsonism and switched to asenapine (titrated over 2 weeks from 5 mg QD to 10 mg twice-daily (BID)).

Within 1 week of initiating valbenazine treatment, caregivers noted an improvement in TD symptoms and no adverse events. After 2 months of valbenazine treatment, the patient had minimal tongue protrusion and lip/jaw movement, resulting in clearer speech, and her arm/hand/finger movements were decreased, leading to improved stability during ambulation (Table 2). She no longer needed a wheelchair and needed less assistance for ADLs such as personal hygiene. She was less isolated and able to go to a restaurant for the first time in months and had started participating in family and church activities. The patient remains stable on valbenazine (80 mg QHS) with no changes to her medication regimen and no reported adverse events (Table 1).

### 2.3. Case 3

A 28-year-old male with moderate ID who lives in a group home had a history of significant comorbid diagnoses, including autism spectrum disorder (ASD), hypertension, hypothyroidism, and GERD (Table 1). Psychiatric diagnoses included excoriation disorder and schizoaffective disorder, bipolar type. In addition, he was anxious, restless, irritable, easily provoked, and physically aggressive and had begun isolating himself from group activities. The patient's medication history included long-term exposure to quetiapine (200/400/200 mg once daily (QD)).

TD symptoms were intermittent chewing movement of the jaw, constant bilateral hand/arm movements (e.g., opening and closing of hands/fingers; finger tapping), repetitive bilateral foot tapping, and truncal rocking (Table 2). The duration of these symptoms is unknown. He was initiated on valbenazine (40 mg QHS for 1 week, then escalated to 80 mg QHS) in February 2018. Due to persistent psychosis, the patient was also tapered off quetiapine and started on clozapine (titrated over 2 weeks to 300 mg daily).

Four days after starting valbenazine treatment, improvement in abnormal movements was noted. After 2 months of valbenazine treatment, the patient had no abnormal jaw movements, minimal to no arm/hand/finger movements or foot tapping, and no truncal movements (Table 2). Caregivers reported that the improvements in movements seemed to result in less isolating, aggressive, and confrontative behavior. He was also more cooperative, was less resistant to caregiver assistance with ADLs, and had increased participation in group home activities. The patient remains stable on valbenazine (80 mg QHS) with no changes to his medication regimen and no reported adverse events.

### 2.4. Case 4

A 61-year-old male with moderate ID who lives in a large group home had a history of multiple significant comorbid diagnoses, including type 2 diabetes, enuresis, hyperlipidemia, hypertension, hypothyroidism, GERD, glaucoma, and tachycardia (Table 1). Psychiatric diagnoses included major depressive disorder, impulse control disorder, obsessive-compulsive disorder, and schizophrenia (paranoid type), for which he had received long-term treatment with multiple typical antipsychotics (chlorpromazine (dose, duration unknown), haloperidol (dose, duration unknown), and thioridazine (dose, duration unknown)) and atypical antipsychotics (clozapine (dose, duration unknown), quetiapine (600 mg QD, duration unknown), and risperidone (25 mg BID, duration unknown)) and was currently receiving treatment with clozapine (50 mg BID and 100 mg QHS).

TD symptoms included intermittent tongue thrusting, chewing motion of the jaw, facial grimacing, and nodding/forward movement of head and neck. In addition, he had frequent eye blinking and constant bilateral hand movement (Table 2). Prior to the onset of his TD symptoms, the patient had been very social and active and was voted “citizen of the year” in his group home, but as his TD symptoms progressed, he had become very agitated, irritable, impatient, and isolated and had stopped participating in group activities. Valbenazine treatment (40 mg QHS for 1 week, then escalated to 80 mg QHS) was initiated in October 2017. No other changes were made to the medication regimen.

Two weeks after starting treatment with valbenazine, abnormal facial movements had improved, and 1 month after initiation, there was no tongue thrusting or chewing motion, minimal facial grimacing and head/neck movement, and normal eye blinking. By 2 months, there were no abnormal hand movements, and he began integrating back into group activities (Table 2). The patient remains stable on valbenazine (80 mg QHS) with no other medication changes and no reported adverse events.

### 2.5. Case 5

A 45-year-old male with mild ID who lives at home with caregivers had a history of significant comorbid diagnoses of hepatitis C, seizures, chronic obstructive pulmonary disease, and paranoid schizophrenia (Table 1). His medication history included prior exposure to the typical antipsychotic, haloperidol (dose, duration unknown), and multiple atypical antipsychotics (aripiprazole, brexpiprazole, olanzapine, quetiapine, risperidone, and ziprasidone (all doses, durations unknown)) and current treatment with clozapine (350 mg daily).

TD symptoms were throughout all body areas, including intermittent lip puckering, jaw movements, facial grimacing, and truncal rocking, as well as constant bilateral shoulder, arm, and hand movement, including piano-playing movements of the fingers (Table 2). His abnormal movements had escalated over the past few months to the point that he had become unable to ambulate and needed wheelchair assistance as well as caregiver assistance with most of his ADLs. In addition, he had become increasingly anxious and hopeless, and had expressed suicidal thoughts. The patient was initiated on valbenazine (40 mg QD for 1 week, then escalated to 80 mg QD) in October 2018. No other changes were made to the medication regimen.

Four days after starting valbenazine treatment, there was a noticeable decrease in abnormal movements, and he was able to ambulate independently with a walker. Two weeks after starting treatment, there were almost no abnormal movements. After 3 weeks of valbenazine, the patient was able to walk independently with a walker, was more independent with his ADLs, and appeared more organized in speech (Table 2). (80 mg QHS) with no changes to other medications and no reported adverse events.

## 3. Discussion

The safety and efficacy of valbenazine, the first approved treatment for TD in adults, have been demonstrated in multiple clinical trials [26, 27, 30, 31]. However, individuals with ID were not included in these trials, as subjects were required to have the capacity to provide informed consent to participate.

This case series presents the clinical characteristics and treatment outcomes of 5 adults (aged 28–63 years) with mild-to-severe ID and TD. All 5 individuals had multiple comorbid psychiatric, behavioral, and other medical conditions; a history of antipsychotic exposure; and abnormal movements affecting the tongue or jaw (*n* = 5), face or eyes (*n* = 3), head (*n* = 1), upper extremities (*n* = 5), lower extremities (*n* = 3), and trunk (*n* = 2), which resulted in diminished ability to speak (*n* = 2), ambulate (*n* = 3), and perform ADLs (*n* = 3). Once-daily valbenazine resulted in marked improvements in TD symptoms within a few weeks of starting treatment, resulting in improvements in daily functioning, demeanor, and social and caregiver interactions. All 5 individuals remained stable after initiation of valbenazine treatment with no adverse events and no TD-related changes to concomitant medications (antipsychotic medications were changed concurrently with valbenazine initiation in 2 individuals due to Parkinsonism and persistent psychosis). The characteristics and outcomes seen in these cases are illustrative of 80-90 additional ID with TD cases seen in our facility.

Challenges in recognizing TD in individuals with ID include a lack of continuity of care in this population, resulting in incomplete or unreliable medical histories, often with an uncertain history of antipsychotic exposure. Furthermore, a lack of known baseline “characteristic” movements for an individual may lead to the assumption that abnormal movements are part of the underlying ID, rather than recognized as possible TD. Individuals with ID and caregivers may be more accepting of adverse events than nondisabled individuals, while others may not be able to report their movement symptoms due to lack of or limited verbal abilities; caregiver input in these situations is crucial. Finally, routine assessments for abnormal movements may be cursory because of the mistaken belief that second-generation antipsychotics do not cause TD.

Given the relatively high likelihood of antipsychotic exposure in the ID population, it is appropriate to screen for TD at every clinical visit through careful monitoring for abnormal movements, particularly in the face, mouth, extremities, and trunk. In addition to careful observation, clinicians should ask questions of both the individual (if they are verbal) and caregiver regarding any abnormal movements or TD-related functional impairments, such as speaking, swallowing, eating, ambulating, social functioning, or other daily activities.

The recent availability of VMAT2 inhibitors has led to increased potential for improved care and quality of life for those affected by TD and a resurgence of interest in improving awareness of TD [32–34]. In this case series, 5 individuals with ID and TD received once-daily valbenazine and experienced marked improvement in their TD symptoms and daily functioning, resulting in increased quality of life for the affected individuals and their caregivers.

## Figures and Tables

**Table 1:** Summary of ID individuals treated with valbenazine for TD.

Case number (gender, age, and living situation)	ID severity	Significant comorbid diagnoses	Medications^a^	
			Antipsychotic	Other
Case 1 (M, 63 yr, group home)	Moderate cognitive impairment; nonverbal, caregiver-dependent for most ADLs	Psychiatric: major depressive disorder Other: arthritis, anemia, GERD, hyperlipidemia, and Parkinson's disease	Prior: quetiapine Current: quetiapine	Prior: atorvastatin, benztropine, carbidopa-levodopa-entacapone, donepezil, duloxetine, escitalopram, memantine, omeprazole, pregabalin, and tramadol Current: atorvastatin, benztropine, carbidopa-levodopa, donepezil, duloxetine, escitalopram, memantine, omeprazole, pregabalin, tramadol, and valbenazine

Case 2 (F, 63 yr, lives at home with caregiver)	Mild cognitive impairment; caregiver-dependent for some ADLs	Psychiatric: schizoaffective disorder (bipolar type) Other: diabetes (type 2), fibromyalgia, GERD, hyperlipidemia, hypertension, hypothyroidism, overactive bladder, and neuropathy	Prior: olanzapine, risperidone Current: asenapine	Prior: benztropine, fluoxetine, gabapentin, levothyroxine, memantine, metformin, mirabegron, N-acetylcysteine, pantoprazole, simvastatin, and topiramate Current: fluoxetine, levothyroxine, memantine, metformin, N-acetylcysteine, oxybutynin, pantoprazole, simvastatin, and valbenazine

Case 3 (M, 28 yr, group home)	Moderate cognitive and physical impairment; caregiver-dependent for mobility and most ADLs	Psychiatric: excoriation disorder, schizoaffective disorder (bipolar type) Other: ASD, hypertension, hypothyroidism, GERD, and seizures	Prior: quetiapine Current: clozapine	Prior: clonazepam, famotidine, lamotrigine, liothyronine, N-acetylcysteine, pindolol, and valproate Current: famotidine, lacosamide, lamotrigine, levothyroxine, lithium, N-acetylcysteine, pindolol, valbenazine, and valproate

Case 4 (M, 61 yr, group home)	Moderate cognitive impairment; caregiver-dependent for some ADLs	Psychiatric: depressive disorder, impulse control disorder, OCD, and schizophrenia (paranoid type) Other: diabetes (type 2), enuresis, hyperlipidemia, hypertension, hypothyroidism, GERD, glaucoma, and tachycardia	Prior: chlorpromazine, clozapine, haloperidol, quetiapine, risperidone, and thioridazine Current: clozapine	Prior: atorvastatin, bethanechol, escitalopram, famotidine, lisinopril, memantine, N-acetylcysteine, and naltrexone Current: atorvastatin, doxazosin, escitalopram, famotidine, glycopyrrolate, latanoprost, levothyroxine, memantine, N-acetylcysteine, pindolol, and valbenazine

Case 5 (M, 45 yr, lives at home with caregiver)	Mild cognitive impairment	Psychiatric: schizophrenia (paranoid type) Other: COPD, hepatitis C, and seizures	Prior: aripiprazole, brexpiprazole, haloperidol, olanzapine, quetiapine, risperidone, and ziprasidone Current: clozapine	Prior: benztropine, buspirone, carbamazepine, citalopram, and fluoxetine Current: duloxetine, gabapentin, and valbenazine

^a^Related to significant comorbid diagnoses. Abbreviations: ADHD, attention-deficit/hyperactivity disorder; ADLs, activities of daily living; ASD, autism spectrum disorder; COPD, chronic obstructive pulmonary disease; F, female; GERD, gastroesophageal reflux disease; ID, intellectual disability; M, male; OCD, obsessive-compulsive disorder; TD, tardive dyskinesia; yr, years.

**Table 2:** TD symptoms and daily functioning in ID individuals before and after treatment with valbenazine.

	TD symptoms/TD-related functional impairment^a^	
	Before valbenazine	After valbenazine (80 mg)
Case 1 (M, 63 yr)	(i) Constant tongue protrusion and intermittent chewing movements of jaw; severe drooling and difficulty swallowing (ii) Excessive, pronounced eye blinking (iii) Bilateral shoulder/hand/finger movement (iv) Constant left foot tapping (v) Instability during standing and ambulating; wheelchair assistance required, and caregiver assistance needed with some ADLs	(i) No tongue protrusion or chewing movements of jaw; able to close mouth; reduced drooling and improved swallowing (ii) Reduced frequency and less pronounced eye blinking (iii) Minimal shoulder/hand/finger movement (iv) No foot tapping (v) Improved stability during standing and ambulating; able to ambulate with walker, and less caregiver assistance needed with ADLs

Case 2 (F, 63 yr)	(i) Repetitive tongue thrusting; lip smacking; chewing movements of mouth/jaw; and unclear speech (ii) Constant bilateral arm/hand/finger movements (iii) Instability during ambulation; some wheelchair assistance required	(i) Minimal tongue thrusting and lip and mouth/jaw movements; clearer speech (ii) Minimal arm/hand/finger movements (iii) Improved stability during ambulation; less assistance required for some ADLs

Case 3 (M, 28 yr)	(i) Intermittent chewing movement of jaw (ii) Constant bilateral arm/hand/finger movements (iii) Repetitive bilateral foot tapping (iv) Truncal rocking	(i) No jaw movements (ii) Minimal to no arm/hand/finger movements (iii) Minimal to no foot tapping (iv) No truncal rocking

Case 4 (M, 61 yr)	(i) Intermittent tongue thrusting, chewing motion of jaw, and facial grimacing (ii) Intermittent nodding/forward movement of head/neck (iii) Frequent eye blinking (iv) Constant bilateral hand movement	(i) No tongue thrusting or chewing motion of jaw, and minimal facial grimacing (ii) Minimal head/neck movement (iii) Normal eye blinking (iv) No hand movement

Case 5 (M, 45 yr)	(i) Intermittent lip puckering, jaw movements and facial grimacing; unclear speech (ii) Constant bilateral shoulder/hand/finger movement (iii) Repetitive bilateral foot tapping (iv) Truncal rocking (v) Instability during ambulation; wheelchair assistance required, and caregiver assistance required for some ADLs	(i) Very minimal lip/jaw movements and no facial grimacing; clearer speech (ii) Minimal shoulder/hand/finger movement (iii) Minimal foot tapping (iv) Minimal truncal rocking (v) Improved stability during ambulation; able to ambulate independently with a cane, and less assistance required for ADLs

^a^In nonverbal or minimally verbal patients, based on caregiver reports and clinician observation during patient visits. Abbreviations: ADLs, activities of daily living; F, female; ID, intellectual disability; M, male; TD, tardive dyskinesia; yr, years.

## Data Availability

Not applicable. No datasets were generated or analyzed.
